# Learning and Using Abstract Words: Evidence from Clinical Populations

**DOI:** 10.1155/2017/8627569

**Published:** 2017-12-19

**Authors:** Maria Luisa Lorusso, Michele Burigo, Alessandro Tavano, Anna Milani, Sara Martelli, Renato Borgatti, Massimo Molteni

**Affiliations:** ^1^Scientific Institute “IRCCS E. Medea”, Bosisio Parini, Lecco, Italy; ^2^Scientific Institute “IRCCS E. Medea”, Pasian di Prato, Udine, Italy

## Abstract

It has been shown that abstract concepts are more difficult to process and are acquired later than concrete concepts. We analysed the percentage of concrete words in the narrative lexicon of individuals with Williams Syndrome (WS) as compared to individuals with Down Syndrome (DS) and typically developing (TD) peers. The cognitive profile of WS is characterized by visual-spatial difficulties, while DS presents with predominant impairments in linguistic abilities. We predicted that if linguistic abilities are crucial to the development and use of an abstract vocabulary, DS participants should display a higher concreteness index than both Williams Syndrome and typically developing individuals. Results confirm this prediction, thus supporting the hypothesis of a crucial role of linguistic processes in abstract language acquisition. Correlation analyses suggest that a maturational link exists between the level of abstractness in narrative production and syntactic comprehension.

## 1. Introduction

In Locke's view [1], concepts originate from the need to categorize and generalize our experience of the world, as it is easier to process a limited number of categories than a great number of individual instances. Traditional psychological theories see concepts as attribute collections (representations) associated with lexical items, which correspond to objects and events in the world (e.g., [2]). These representations would be extracted through perceptual and linguistic experience derived from our interaction with the world [3–5]. In Quinn and Eimas' view [6], concepts develop gradually through a process of quantitative enrichment, based on perceptual learning. Infants would form a category representation for a set of entities (e.g., animal or animal-like) by encountering various individual examples over time and extracting common physical attributes [7, 8]. Other authors postulate the existence of supplementary processes integrating perceptual analysis. Mandler and McDonough [9, 10], in what is usually referred to as a dual-process framework, suggest that concepts are initially formed by “perceptual schemas” that define what the objects that fall into each conceptual category would generally look like. Subsequently, a second process would produce “image schemas” by extracting dynamic information such as motion, sound, and function (i.e., categories based on meaning). Other authors (e.g., [11]) suggest that perceptual learning is complemented by inferential mechanisms giving information about function, intention, and so forth.

All approaches to conceptual development in children consider sensory (primarily visual) experience as fundamental to build and represent concepts. Even when inferential mechanisms are called for, they are usually assumed to apply to directly experienced elements in the attempt to find more general categories that include them. However, abstract concepts do not meet such requirements, because they do not stand for any perceivable object. How are concepts such as* knowledge, creativity, justice, science, and happiness* formed, if there are no concrete referents in the world to serve as examples?

In most studies, abstract knowledge has been considered mainly by opposition to concrete knowledge, focusing on the so-called* concreteness effect* (i.e., the greater ease and speed in processing concrete concepts as compared to abstract concepts) [12–16] or on different contents and network structures characterizing the two classes of words (e.g., [16, 17]). However, no convergence has been reached about how abstract concepts are learned and represented.

From an acquisition viewpoint, a set of mechanisms proposed in the literature might be specifically relevant for abstract concept formation. For instance, “cross-situational learning” [18–21] refers to the situation where a child hears a new word and he/she is assumed to activate a number of potential meanings for that word from the nonlinguistic context of the utterance containing that word. When the same word is heard in several different utterance contexts, the child can intersect the corresponding sets to find which meaning is consistent across the different occurrences of that word. Abstract concept acquisition would mainly depend on the system's sensitivity to input distributional features such as occurrence statistics [22]. Syntactic bootstrapping mechanisms [19, 23, 24] may also play a role as it has been shown that syntactic structure helps narrowing the search space for verb meaning. Another set of theories, on the contrary, emphasize the role of sensory-motor experience also in the acquisition of abstract knowledge, in a sort of simulated or situated experience involving abstract concepts [25–27]. Neuroimaging studies seem to reflect the same multiplicity of views. A recent meta-analysis of 19 neuroimaging studies compared the processing of abstract and concrete concepts across various tasks and found that left inferior frontal, superior temporal, and middle temporal cortices were more active for abstract than for concrete concepts across studies [28]. Concrete concepts, by contrast, activated the posterior cingulate, precuneus, fusiform gyrus, and parahippocampal gyrus. This suggested that abstract concepts rely on verbal systems, whereas concrete concepts rely on perceptual systems supporting mental imagery (see also [29]), contrasting with other studies showing activation of motor areas or, more recently, distributed neural systems representing concept-specific content [30, 31] during the processing of abstract expressions. Many of these studies are thus consistent with the prediction of the dual coding model showing that the right hemisphere is indeed more activated by concrete than abstract words [32] or that concrete versus abstract words activate a distributed, bilateral semantic memory system [33, 34]. A similar dissociation is reported in studies of patients showing greater impairments for concrete words than abstract words following bilateral or unilateral ventral temporal lesions (e.g., [35]) or showing greater impairment for abstract words following left perisylvian damage (e.g., [36]).

Children with Down Syndrome (DS) and children with Williams Syndrome (WS) offer a unique opportunity to study the development of abstract versus concrete language. Williams Syndrome (WS) is a rare, genetically based neurodevelopmental disorder caused by the deletion of one copy of a small set of genes on chromosome 7 (7q11.23). Children with WS are characterized by major visual-spatial and constructional impairments [37, 38] accompanied by close-to-normal, though not fully intact linguistic skills [39, 40]. More specifically, no deficits in phonetic and phonological skills are usually reported, with good short-term memory skills [39], and their speech is described as fluid [41] and lexically rich (e.g., [42], but see [43, 44], or [45] for more detailed reviews of the neuropsychological data). The frequent use of uncommon terms is often reported (e.g., [46]), although no systematic distinction has been made between abstract and concrete ones. Mervis and Morris [47] actually reported that children with WS present with a specific weakness in the use of relational terms, not limited to spatial terms and thus not directly consequent to their impairment in spatial abilities. The authors also suggest that this impairment may be linked to the structural abnormalities found [45] for WS in what Walsh [48] called the “common magnitude system,” an area partially located in the inferior parietal cortex and controlling spatial, temporal, and quantitative processing.

Down Syndrome (DS) is a chromosome abnormality caused by trisomy of chromosome 21 and children affected by this syndrome are mainly characterized by severe linguistic impairments, with relatively spared visual-spatial and visual memory skills [49–51]. The delay in linguistic development appears to be more evident for morphological and syntactic skills than for lexical-semantic skills [42, 52, 53], although a general linguistic impairment is usually reported [54]. Among lexical skills, a dissociation has been reported between receptive and productive vocabulary, with the former being relatively more preserved than the latter [55]. Nonetheless, even at the receptive level, that is usually described as a relative strength for individuals with DS, difficulties have been observed with the comprehension of abstract relational terms such as* between*,* separated*, and* equal* [56]. Chapman and colleagues [57] further showed that adolescents with DS produce a lower number of total words, and a more limited variety of different words, if compared to TD controls matched on mental age. This reduction is not accounted for by limitations in language production overall, since adolescents with DS produce even more utterances (yet, less clearly intelligible) in the time unit as compared to controls. In spite of their length (MLU mostly above 3 in the adolescents who participated in the study), utterances in the DS group were characterized by a large number of omissions, essentially regarding function words [57]. No direct comparison in productive lexicon between concrete and abstract nouns is reported in any of the above studies, but Chapman et al.'s [58] finding of the predictive value of receptive vocabulary with respect to productive vocabulary suggests that concrete words are likely to be more represented than abstract ones and that these differences may be larger for individuals with DS than for the typically developing population.

Altogether, then, children with DS are characterized by predominant impairments in linguistic computational skills but relatively preserved visual-spatial abilities, while children with WS present with visual-spatial impairments but better preserved linguistic skills [59, 60]. Both groups appear to have more impaired comprehension and production of abstract (use of relational terms in the above-mentioned studies) vocabulary as compared to concrete vocabulary; however, no systematic comparison of the two types of lexical production (especially if focusing on spontaneous production rather than naming or recalling and on open-class abstract language production rather than function words) between the two clinical populations has been conducted, to our knowledge.

Beyond descriptive aims, the neuroanatomical features of the two populations allow testing the role of different neural networks suggested to be involved in the processing of abstract and concrete words. In fact, Williams Syndrome was shown to have decreased overall brain and cerebral volumes, relative preservation of cerebellar and superior temporal gyrus (STG) volumes, and a reduction of right occipital areas, resulting in a greater ratio of frontal to posterior (parietal and occipital) tissue [61]. On the other hand, Down Syndrome neuroanatomy is characterized by reductions in gray matter (GM) tissue in the cerebellum, cingulate gyrus, left medial frontal lobe, and right middle/superior temporal gyrus [62].

It was shown in previous studies [63] that individuals with WS are as sensitive to phonological similarity and concreteness effects in memory tasks as typically developing children. This suggests that the acquisition of abstract terms in WS follows typical pathways (although with slower rhythms), in spite of limitations in visual-spatial skills. Based on such data, together with the expectations that descend from the neuroanatomical and neurofunctional characteristics of the two clinical populations, we could predict that children with WS should outperform children with DS matched on mental age in the use of abstract terms and at the same time show a more limited concrete concept repertoire. Since, however, neuroimaging data and studies of brain-damaged patients are almost exclusively based on analyses of vocabulary comprehension, lexical decision, word repetition, or word retrieval, with few existing studies on lexical production, a study on vocabulary production could add new evidence to our understanding of the neural and cognitive substrates of abstract thinking. Moreover, the use of a highly ecological testing procedure tapping narrative production could provide valuable information with respect to everyday language use in meaningful contexts for children and for individuals with intellectual disabilities. Indeed, picture story retellings have been widely employed to trace the trajectories of language development in a variety of typically developing and clinical populations (see, e.g., [64]), and they are considered to be particularly valuable to elicit descriptions of mental states and intentions [65, 66], which can prove very helpful for the assessment of abstract vocabulary.

## 2. Methods

### 2.1. Experimental Design

Since experience is also likely to be involved in conceptual acquisition, we aimed at matching WS and DS participants on mental age (MA) (calculated on the basis of full-scale IQ on the Wechsler scales) as well as on chronological age (CA). Further, each of the clinical groups was compared to a younger group of typically developing children (TD) of the same MA. In this comparison, WS and DS children should be more impaired than TD children in visual-spatial and linguistic skills, respectively. Such a contrast allows a tighter control of atypical development trajectories in the neuropsychological development of clinical groups.

No simultaneous match on both CA and MA between children with DS and WS was possible though, due to a generally greater intellectual impairment in the DS group as compared to the WS group. Therefore, we performed the following four different comparisons:

- DS and WS participants matched on CA
- DS and WS participants matched on MA
- DS and TD participants matched on MA
- WS and TD participants matched on MA

The study was approved by the institute's Ethical Committee according to standards of Helsinki Declaration (1964).

### 2.2. Participants

Seventeen participants with Down Syndrome (mean age 12.8, range 8 to 18.4 years) and 13 participants with Williams Syndrome (mean age 13.8, range 8.2 to 18.7 years) participated in this study (see Tables 1 and 2 for details).

Participants with WS were in charge at the Research Institute “E. Medea” in Bosisio Parini, Italy, and had been positively diagnosed by a geneticist. Participants with a similarly clinically confirmed diagnosis of DS were recruited from the Research Institute “E. Medea” in San Vito al Tagliamento, Italy, and through local patient welfare associations. All children attended mainstream schools but followed individualized academic curricula. A group of typically developing children was recruited in local primary schools. Teachers described their development as typical. All participants were native Italian speakers. Informed consent was signed by the parents of all participants.

Data were collected on a number of different variables. In order to allow matching of the two groups (WS and DS) on CA and MA; full-scale IQs as measured by the WISC-R scale [67, 68] were collected from individual records (in case the test had been administered more than 2 years before recruitment for the present study, a new test session was organized). MA was calculated on the base of ad hoc norms [67, 68].

The mean scores for the WS group were CA = 13.3, MA = 6.9. Scores for the DS group were CA = 12.7, MA = 6.11. Since no data were available on the control group's IQ scores, MA was assumed to be equivalent to CA.

Linguistic performance data were collected using four tests: Passive Vocabulary (an Italian adaptation of the British Picture Vocabulary Scale (BPVS) [69]), Naming [70], Sentence Repetition [71, 72], and syntactic comprehension (Token Test, reduced version in 21 items [70, 73]). Italian adaptation and norms were taken from Fabbro [74]. All results were expressed as age-equivalent scores, based on standard norms. The scores of the BPVS and the Naming test were subsequently averaged into an index of lexical-semantic Competence, whereas scores from Sentence Repetition and Token test were averaged into an index of syntactic competence. Further, an average of BPVS and Token test scores was calculated to express Linguistic comprehension skills, while the average of Naming and Sentence Repetition scores was meant to express linguistic production skills.

The characteristics of the two groups of participants are reported in Tables 1 and 2.

### 2.3. Materials and Procedure

Concreteness indices were extracted from story-telling recordings. Narratives were elicited using the wordless picture book “Frog, Where Are You?” [75], already successfully used to tap into typical and atypical complex language development [76, 77]. Participants were first informed that they would be asked to tell a story and then invited to look through the entire booklet and finally to tell the story, again while looking at the pictures. The exact instructions provided by the experimenter were as follows: “here is a book: this book tells a story about a boy, a dog and a frog (pointing to the picture on the cover). First I want you to look at all the pictures. Pay attention to each picture, try to understand what happens, afterwards you will see the pictures again and you will tell the story” ([76]; p. 22). Participants had no time limit to look through the story. All stories were recorded on a digital recorder, transcribed, and finally coded in CHILDES format [78]. In the first analysis closed-class words, adverbs and auxiliaries were excluded from the story, while verbs were all coded in their infinitival form.

The whole list of words used by the participants was subsequently rated by 20 adult native speakers for concreteness, on 1 (totally abstract) to 7 (totally concrete) scale. Two sets of words were created using a dichotomized code where the words rated 4 or lower were treated as abstract while words rated higher than 4 were considered to be concrete. Finally, a general index of concreteness of language was calculated by expressing the percentage of concrete words over the total number of words (e.g., a subject that produced 7 concrete words over a total of 10 words had a percentage of 70%). This procedure allows controlling for the discrepancy in number of uttered words between the DS group and the WS group, as individuals with Williams Syndrome are generally more talkative with respect to individuals with Down Syndrome. According to this measure, participants with lower indices present a more extensive use of abstract words than participants with higher indices.

First, we used two-tailed *t*-tests to compare WS to DS participants matched one-to-one on the basis of chronological age (±6 months). Then, a second analysis matched participants one-to-one on MA (±6 months). Finally, each atypically developing group was tested against typically developing peers. A correlation analysis using Pearson's *r* was also carried out in order to investigate the relationship between linguistic components measured by the linguistics tests and concreteness indices. Throughout, *p* values ≤ 0.05 were considered significant.

## 3. Results

Ten subjects from each atypically developing group were included in the first analysis (see Tables 1 and 2, column a). DS participants had a mean CA of 14.1, SD = 3.1, range 8 to 17 years; WS had a mean CA of 13.7, SD = 2.8, range 8.2 to 17.6 years. Performance of the WS group was significantly, or marginally significantly, better on all indices compared to performance in the DS group: lexical-semantic, *t*(18) = 1.91, *p* = 0.07, *d* = 1.294; syntactic, *t*(18) = 3.07, *p* = 0.01, *d* = 1.665; comprehension *t*(18) = 2.01, *p* = 0.06, *d* = 1.251; production, *t*(18) = 2.21, *p* = 0.04, *d* = 1.401. No correction for multiple comparisons had been applied in this analysis, being all variables correlated and being all differences expected based on the characteristics of the two clinical groups. Moreover, WS participants had significantly lower concreteness indices (M = 65.02, SD = 4.65) than DS participants (M = 72.69, SD = 5.89) (*t*(18) = −2.31, *p* = 0.033, *d* = 1.445).

In order to control for the difference in general cognitive skills of participants with Down Syndrome with respect to participants with Williams Syndrome, the second analysis matched participants of the two groups one-to-one on MA (±6 months). Ten participants were selected from each group (see Tables 1 and 2, column b) (DS: mean MA = 6.6, SD = 1.3, range 4.3 to 8.3 years; WS: mean MA = 6.6, SD = 1.4, range 3.8 to 8.2 years). In this case, performance of the WS group compared to the DS group was significantly better on syntactic indices only: *t*(18) = 2.67, *p* = 0.02, *d* = 1.794. The pairwise comparison showed that WS participants had a significantly lower concreteness index (M = 65.25, SD = 6.3) than DS participants (M = 73.46, SD = 4.57): *t*(18) = −2.82, *p* = 0.01, *d* = 1.492.

In order to test whether the differences in the proportion of concrete words used by the two groups in narrative production could be ascribed to specific neuropsychological characteristics of atypically developing populations, however unrelated to the discrepancy between linguistic and visual-spatial abilities, DS and WS participants were separately compared to typically developing children of a similar mental age. As a larger number of narratives had been collected from TD children, it was possible to include all DS (except one) and WS participants in the analyses.

In the first analysis, 16 DS participants (mean age 13.4, DS = 3.5, range 8 to 18.3 years) were compared with 16 TD children (mean age 6, DS = 1.3, range 4.2 to 8 years). The pairwise comparison revealed that DS participants have significantly higher concreteness indices (M = 73.41, SD = 6.53) than TD children (M = 68.02, SD = 8.9) (*t*(30) = 2.65, *p* = 0.01, *d* = .69).

The second analysis compared 13 WS participants (mean age 12.9, DS = 4, range 7.11 to 18.5 years) with 13 TD children (mean age 6.4, DS = 2, range 4.1 to 9.42 years). This comparison did not show any significant difference between the two groups (*t*(24) = −0.6, n.s.).

A correlation analysis was also carried out in order to investigate the relationship between linguistic components measured by linguistic tests and general concreteness indices. Pearson correlation indices were computed for linguistic variables including lexical-semantic components, syntactic components, comprehension, and production. Separate analyses for DS, WS, and TD participants did not reveal any correlation with concreteness (possibly due to limited number of participants). However, when including all participants in the analysis (*n* = 60), a negative correlation emerges between concreteness and both comprehension (*r* = −.315; *p* = 0.008) and syntactic components (*r* = −.248; *p* = 0.041). No other correlations reached statistical significance.

## 4. Discussion

The aim of the present study was to compare lexical concreteness levels in the narratives of clinical populations known to have particularly marked deficits in either language (Down Syndrome) or visual-spatial skills (Williams Syndrome), in order to determine whether linguistic as opposed to visual-spatial processes play a crucial role in the acquisition of abstract knowledge. For this purpose, story-telling recordings produced by individuals with Williams Syndrome and by individuals with Down Syndrome were compared with respect to the proportion of concrete and abstract words. Statistical analyses revealed that WS participants present, overall, a more extensive use of abstract words than DS participants, whether they are of comparable chronological age or of comparable mental age. This finding favors the hypothesis that the development of abstract knowledge is based on linguistic skills, such as the extraction of regularities from linguistic input [22]. Clearly, an advantage for individuals with Williams Syndrome in the use of abstract language is only possible if their (weak) [37] visual-spatial system is not crucially or only marginally involved in the acquisition of abstract concepts. On the other hand, the idea that abstract concepts are learned through the process of extracting statistical regularities from language is confirmed by the fact that participants with DS, who are characterized by a more severe linguistic impairment [52], present a more concrete language with respect to both WS participants (either paired on CA or MA) and TD children of the same general MA (thus presumably expressing an average of slightly worse visual-spatial abilities and slightly better linguistic skills). This extends previous findings about specific impairments in DS concerning function words [57] or relational terms [56] to the more general class of abstract words.

The tendency of individuals with Williams Syndrome to learn and use low-frequency terms (e.g., [46]) may contribute to the differences described in the present study, as abstract words are in general less frequent than concrete ones. However, the atypical frequency effects observed in children with Williams Syndrome have been explained by some authors as a particular sensitivity to the morphological formal structure of words [79], by others as a dissociation of enhanced phonological representations and poor lexical-semantic representations [80, 81], or a dissociation of intact combinatory and rule-governed grammatical skills and poor fine-grained lexical-semantic specifications [82]. Laing et al. [63] addressed this issue as related to verbal memory and concluded that the processing of semantic aspects of vocabulary is rather well developed in individuals with WS. The present results, together with these studies, appear to support a linguistic explanation for the advantage of Williams Syndrome participants in the use of abstract words, even if the exact contribution of formal and semantic aspects of abstract word acquisition (and use) should be further investigated.

We would not argue that visual-spatial abilities do not play any role in building and processing abstract concepts. Sensory-motor experiences associated with the acquisition and representation of abstract concepts might be retrieved and reactivated (or simulated, according to some conceptualizations of embodied cognition) any time the concept is accessed [83, 84]. However, impairment at the visual-spatial level does not significantly hinder the acquisition or use of abstract concepts, as demonstrated by the absence of significant differences between individuals with Williams Syndrome and typically developing children of the same mental age. This also confirms, albeit indirectly so, the predominant involvement of left anterior areas (the most preserved in WS [61]), and possibly of the cerebellum, in the development of abstract language, whereas better functioning right hemisphere structures as found in DS [62] seem to be less crucial in these terms. Such a pattern of results is consistent with Paivio's dual coding theory [12, 13] and with other studies suggesting that abstract concepts are mainly represented in the left hemisphere [28, 29, 36].

Finally, the analysis of correlational patterns provides some insight into the nature of linguistic skills and processes involved in abstract concept acquisition and use. Although emerging only when pooling all participants together (a likely statistical power issue), it appears that the linguistic skills more directly involved in the acquisition of abstract concepts are to be found in the* syntactic* rather than in the* lexical-semantic* components and in* comprehension* rather than in* production*. This is compatible with the hypothesis suggesting that the crucial process of the extraction of regularities in input language can also be seen as the basis of syntactic competence (e.g., [19, 22, 85]). Moreover, it is consistent with previous studies showing that comprehension abilities are predictive of production performance [58]. The whole set of results, thus, points to linguistic abilities, including those that involve the analysis of the syntactic structure in input, as an important basis for building and representing abstract knowledge. This also appears consistent with recent results from various types of studies [86, 87].

## 5. Conclusions

One of the assumptions of the study was that specific difficulties in either the linguistic or the visual-spatial domain would interfere with the acquisition of abstract concepts and that this disadvantage would be evident in the linguistic production of children with genetic syndromes especially affecting the verbal or the visual-spatial domain. What is clear from the outcomes of the study is that the interference from linguistic impairments is significant, in contrast to interference from visual-spatial impairments. Whether this interference occurs in the very early stages of conceptual development, or at a later stage of abstract knowledge development, such as the construction of semantic networks including the abstract nodes or the reactivation of previously stored information, is an interesting question that may deserve further investigation.

## Figures and Tables

**Table 1:** Characteristics of participants with Williams Syndrome.

WS	CA	MA	IQ	L-Sem	Synt	Comp	Prod	a	b
WS1	16.11	8.3	49	11.5	5.26	9	8.1	*∗*	*∗*
WS2	13.4	5.10	44	5	4.75	4.25	5.5	*∗*	*∗*
WS3	18.7	7.2	48	9.5	3.98	8.12	5.35		*∗*
WS4	8.2	4	49	9	5.37	8.25	6.12	*∗*	*∗*
WS5	10.7	5.5	52	5.12	3.87	5	4	*∗*	*∗*
WS6	12	7.2	60	11.5	7.62	11.5	7.62	*∗*	*∗*
WS7	15.7	7.7	50	8.12	6.62	9.25	5.5	*∗*	*∗*
WS8	17.6	9.5	54	9.2	9.46	12	6.71	*∗*	
WS9	13.9	6.4	47	8.4	4.25	8.25	4.37	*∗*	
WS10	11.6	8.2	71	9	5.62	8	6.62		*∗*
WS11	15.1	7.6	50	10.8	6.37	10.25	6.87	*∗*	
WS12	11.6	5.7	49	3.8	5.5	3.62	5.62		*∗*
WS13	14.4	7.1	51	10.2	5.12	7.75	7.62	*∗*	*∗*

*Mean*	13.7	6.8	51.8	8.5	5.7	8.1	6.16		
*SD*	2.9	1.5	6.9	2.5	1.5	2.5	1.2		

CA = chronological age; MA = mental age; IQ = intelligence quotient; L-Sem = lexical-semantic competence (age equivalent); Synt = syntactic competence (age equivalent); Comp = linguistic comprehension skills (age equivalent); Prod = linguistic production skills (age equivalent); a = participants selected for the match on CA; b = participants selected for the match on MA.

**Table 2:** Characteristics of participants with Down Syndrome.

DS	CA	MA	IQ	L-Sem	Synt	Comp	Prod	a	b
DS1	14.1	6.11	49	11	4.75	8.12	7.62		*∗*
DS2	8	5.9	72	3.37	3.37	3.62	3.12	*∗*	
DS3	14.4	6	43	4.62	4	4.62	4	*∗*	*∗*
DS4	9.3	5.3	57	9	3.5	5.75	6.75		*∗*
DS5	8.10	4.6	51	6.2	3.75	5.87	4.12		*∗*
DS6	17	8.4	49	4.62	3	4.37	3.25	*∗*	*∗*
DS7	15.10	7.9	49	8.5	3.25	5.62	6.12	*∗*	*∗*
DS8	15.7	5.11	40	8	4.62	6.75	5.87	*∗*	
DS9	13	6.6	50	5	3.37	4.37	4		
DS10	18.4	7.8	42	7.5	3.25	5.25	5.5	*∗*	
DS11	9.10	4.9	48	5.5	3.62	4.75	4.37		
DS12	10.8	4.10	45	5.5	3.12	4.25	4.37	*∗*	
DS13	12.1	4.4	36	6.5	4.25	6.62	4.12	*∗*	
DS14	16.11	8.3	49	8.12	4	10.5	4.87	*∗*	*∗*
DS15	8.8	6.1	70	10.5	3.62	7.25	6.87		*∗*
DS16	13.6	6.11	51	5.12	4.62	5.75	4	*∗*	*∗*
DS17	17.5	6.7	44	4.5	3.25	3.87	3.87		*∗*

Mean	13	6.13	49.7	6,4	3,7	5,5	4,7		
SD	3.4	1.3	9.4	2,4	0,56	1,8	1,4		

CA = chronological age; MA = mental age; IQ = intelligence quotient; L-Sem = lexical-semantic competence (age equivalent); Synt = syntactic competence (age equivalent); Comp = linguistic comprehension skills (age equivalent); Prod = linguistic production skills (age equivalent); a = participants selected for the match on CA; b = participants selected for the match on MA.
